# Alternative isoform expression of key thermogenic genes in human beige adipocytes

**DOI:** 10.3389/fendo.2024.1395750

**Published:** 2024-05-24

**Authors:** Sarah Hazell Pickering, Mohamed Abdelhalim, Philippe Collas, Nolwenn Briand

**Affiliations:** ^1^ Department of Molecular Medicine, Institute of Basic Medical Sciences, Faculty of Medicine, University of Oslo, Oslo, Norway; ^2^ Department of Immunology and Transfusion Medicine, Oslo University Hospital, Oslo, Norway

**Keywords:** beige adipocyte, human thermogenic adipocytes, adipose differentiation, transcriptomics, alternative transcript isoforms, alternative splicing, human adipose stem cells (hASCs)

## Abstract

**Background:**

The beneficial effect of thermogenic adipocytes in maintaining body weight and protecting against metabolic disorders has raised interest in understanding the regulatory mechanisms defining white and beige adipocyte identity. Although alternative splicing has been shown to propagate adipose browning signals in mice, this has yet to be thoroughly investigated in human adipocytes.

**Methods:**

We performed parallel white and beige adipogenic differentiation using primary adipose stem cells from 6 unrelated healthy subjects and assessed differential gene and isoform expression in mature adipocytes by RNA sequencing.

**Results:**

We find 777 exon junctions with robust differential usage between white and beige adipocytes in all 6 subjects, mapping to 562 genes. Importantly, only 10% of these differentially spliced genes are also differentially expressed, indicating that alternative splicing constitutes an additional layer of gene expression regulation during beige adipocyte differentiation. Functional classification of alternative isoforms points to a gain of function for key thermogenic transcription factors such as *PPARG* and *CITED1*, and enzymes such as *PEMT*, or *LPIN1*. We find that a large majority of the splice variants arise from differential TSS usage, with beige-specific TSSs being enriched for PPARγ and MED1 binding compared to white-specific TSSs. Finally, we validate beige specific isoform expression at the protein level for two thermogenic regulators, PPARγ and PEMT.

**Discussion:**

These results suggest that differential isoform expression through alternative TSS usage is an important regulatory mechanism for human adipocyte thermogenic specification.

## 1 Introduction

Adipose tissue has become a major research focus because of its capacity to store energy in the form of lipids in white adipocytes and dissipate energy by heat in brown and beige (thermogenic) adipocytes. Thermogenic adipose tissue is found in human adults (1–3) as brown and beige depots in posterior regions of the neck, thorax and abdomen, and as beige adipocytes interspersed within white fat depots (4, 5). White adipocytes, which make up the majority of adipocytes in white fat depots, store energy in the form of lipids, while brown and beige adipocytes can dissipate energy as heat by uncoupling mitochondrial respiration from ATP production, or by activating other ATP-consuming futile cycles (6–8). Beige adipocytes arise in white depots by differentiation of progenitors and by conversion from a white phenotype in response to cold, or to PPARγ-agonist treatment. Thermogenic adipocyte activity correlates with increased insulin sensitivity and decreased incidence of obesity and type-2 diabetes in humans (9), thus elucidating the regulatory mechanisms defining white vs beige adipocyte specification may provide new targets for treating metabolic diseases.

Alternative splicing increases transcriptome and proteome diversity by modulating the combination of exons expressed from a single gene, thereby regulating diverse processes such as cell differentiation and signal transduction (10). Whole or parts of exons may be substituted by selection of the 5’ or 3’ splice sites by the key components of the spliceosome, or by accessory splicing factors which recognize weak signals in the surrounding RNA sequence (11). Alternative splicing events are classified into several types: exon skipping, mutually exclusive exons, intron retention and alternative 5’ and 3’ splice site usage (12). Sometimes included in this list are alternative first exons, which can be generated by alternative promoters or transcription start sites (TSS) (13, 14). Differences in alternative splicing between conditions can be assessed in short read RNA-seq by algorithms inferring differential splicing by assigning reads to isoforms found in a transcriptome, or by directly detecting exon-exon junctions (15, 16). Alternative splicing may influence protein function, localization or degradation, and the 5’ and 3’ untranslated regions of mRNAs also affect their secondary structure, export and translation efficiency (17). Thus, assigning functionality to the products of alternative splicing is still challenging. However, recent developments such as the establishment of protein isoform annotation databases and associated scoring tools now provide insights into variant functionality (18–21).

Alternative splicing has emerged as a key mechanism regulating white and thermogenic adipogenesis. Splice variants in nuclear corepressors and nuclear transcription factors have been shown to directly regulate adipogenesis (22, 23). In addition, perturbation of splicing factors in mice affects thermogenesis in brown (24, 25) and white adipose depots (26, 27). Recent evidence has suggested a potential role for splicing in the browning process of human adipocytes (28). Indeed, transcriptomic analysis of adipose tissue from patients with pheochromocytoma tumors revealed a global downregulation of splicing factors’ expression, a trend confirmed in cold-exposed mouse inguinal adipose tissue (28). However, the isoform specific gene expression program characterizing thermogenic adipocytes has not been comprehensively investigated.

Here, we analyze differential gene and transcript expression in white and beige adipocytes differentiated from 6 human primary adipose stem cell (ASC) lines. We unveil a high-confidence set of differentially spliced genes, including in key thermogenic regulators for which we validate beige-specific isoform expression at the protein level. Our results suggest that the regulation of isoform-specific expression is an important regulatory mechanism for the thermogenic function of human adipocytes.

## 2 Materials and methods

### 2.1 Cell culture

Primary ASCs were isolated from subcutaneous fat obtained by liposuction from six unrelated female donors (**Supplementary Table 1**) after informed consent (approval by the Regional Committee for Research Ethics for Southern Norway, REK 2013/2102 and 2018-660). ASCs were cultured in DMEM/F12 (17.5 mM glucose) with 10% fetal calf serum and 20 ng/ml basic fibroblast growth factor (proliferation medium). Upon confluency, fibroblast growth factor was removed, and cells cultured for 72 h in DMEM/F12 (17.5 mM glucose) with 10% fetal calf serum (basal medium) before induction of differentiation. For white adipose differentiation, ASCs were induced with a cocktail of 0.5 µM 1-methyl-3 isobutyl xanthine, 1 µM dexamethasone, 10 µg/ml insulin and 200 µM indomethacin in basal medium. Differentiation media was renewed every 3 days until day 9, after which cells were maintained in DMEM/F12 (17.5 mM glucose) with 10% fetal calf serum and 10 µg/ml insulin. For beige adipose differentiation, media were supplemented with 1 µM Rosiglitazone (Sigma, R2408) until day 15. Samples were harvested 15 days after induction. All differentiation experiments were done in at least three biological replicates between passage 3 and 8.

### 2.2 Microscopy and image analysis

Cells were differentiated for 15 days on 12-mm diameter coverslips in 24-well plates. Cells were washed 3 times with PBS before fixation in 4% paraformaldehyde for 10 min. Cells were then incubated in Bodipy (1µg/ml, Invitrogen D3922) for 15 min and washed 3 times in PBS before mounting in DAKO Fluorescence Mounting Medium (S3023, Agilent). Images were acquired on an IX81 microscope (Olympus) fitted with epifluorescence, an 100× 1.4 NA objective mounted on a piezo drive, and a DeltaVision personalDV (Applied Precision, Ltd.) imaging station. The Bodipy surface per field was quantified on threshold-adjusted images using ImageJ software.

### 2.3 Immunoblotting

Proteins were resolved by gradient 4-20% SDS–PAGE, transferred onto nitrocellulose membranes (BioRad) and blocked with 5% BSA. Membranes were incubated for 1h at room temperature or overnight at 4°C using the following antibodies: Perilipin1 (Progen, GP29), CITED1 (Novus, H00004435-M03), UCP1 (Abcam, 23841), PPARγ (Thermofisher, MA5-14889), PEMT (Novus, NBP1-59580), and γTubulin (Sigma, T5326). Proteins were visualized using IRDye-800-, IRDye-680-, or HRP-coupled secondary antibodies. Bands were quantified by densitometry (Image Lab; BioRad) using γTubulin for normalization. Uncropped membranes are presented as **Supplementary Data 1**-**4**.

### 2.4 RNA-sequencing and gene expression analysis

RNA sequencing (RNA-seq) was done in biological triplicates for 6 independent donors. Total RNA was isolated from samples harvested at differentiation endpoint (day 15) using the RNeasy kit (QIAGEN). PolyA-selected RNA was sequenced from paired-end libraries (TruSeq Stranded mRNA kit; Illumina) using Novaseq platform (Illumina). Reads were filtered with fastp, aligned to the hg38 genome (GENCODE 32 annotation) with hisat2, and counted using featureCounts (**Supplementary Table 2**). Low abundance genes were filtered using filterByExpr from edgeR and then normalized using the trimmed mean of M values method (29). Beige and white gene expression was compared pairwise for each donor using the robust eBayes method with limma-voom adjustment (30). Less than 100 genes changed according to the fraction of reads assigned to genes (adjusted p-value < 0.01), when this was included as a continuous variable in the model.

### 2.5 RT-qPCR and semi-quantitative PCR

RNA was isolated using RNeasy kit (QIAGEN) and 1 µg was used for cDNA synthesis using High-Capacity cDNA Reverse Transcription Kit (ThermoFisher). RT-PCR was done using IQ SYBR green (Bio-Rad Laboratories) with *SF3A1* as a reference gene. PCR conditions were 95°C for 3 min and 40 cycles of 95°C for 30 s, 60°C for 30 s, and 72°C for 20 s. PCR primers are listed in **Supplementary Table 3**.

### 2.6 Alternative splicing analysis

Reads were aligned to the hg38 genome with STAR using the GENCODE 32 reference annotation. ENCODE options from the STAR manual were used as well as –outSAMstrandField intronMotif –outSAMtype BAM Unsorted (**Supplementary Table 2**). Split reads with at least 6 bp of anchor sequence and an intron length between 20 bp and 1 Mb were extracted using regtools (31). Introns sharing splice sites were clustered using LeafCutter’s leafcutter_cluster_regtools.py script, requiring at least 30 reads per library supporting each cluster for introns of up to 1 Mb in size.

Differences in intron excision were tested between white and beige triplicates with donor as a confounding variable. Strict prefiltering required that an intron was found in at least 14 (of 36) libraries and that clusters had at least 12 libraries per condition with 20 or more spliced reads. The differential_splicing function from LeafCutter R package version 0.2.9 was used to implement the Dirichlet-multinomial generalized linear model (15). LeafCutter assigns a ΔPSI to each intron and a p-value to each cluster, which contains introns that share splice sites and thus belong to alternative isoforms of that gene or genes. Effect size and cluster significance output files were merged into a single table containing both ΔPSI and cluster p-value (**Supplementary Table 4**). LeafCutter p-values are adjusted by FDR.

### 2.7 Annotation of introns

Introns were assigned to transcripts, with the priority for annotations: GENCODE 32 > RefSeq GCF_000001405.40-RS_2023_10 > FANTOM CAT robust (32). In other words, only if an intron was missing from the GENCODE annotation was it compared to the RefSeq annotation and so on. Normalized TRIFID prediction scores were downloaded for the GENCODE 37 and RefSeq110 annotations. Where multiple transcripts were possible for a single intron excision event, TRIFID scores were averaged, but if no score was found -0.1 was used. To calculate a TRIFID difference, the top two intron-excision events were compared for clusters with at least one significant junction (| ΔPSI | > 0.1 & p.adjust < 0.05) (**Supplementary Table 5**).

For ChIP-Seq profiles at TSSs, for each significant exon-exon junction, the most upstream annotated TSS was selected to avoid oversampling of junctions. For Unibind differential enrichment (beige vs white) at TSSs, overlapping -2 kb/+0.5 kb windows were merged.

### 2.8 Chromatin immunoprecipitation-seq

Day 15 differentiated white and beige samples were trypsinized, resuspended in HBSS, 0.5% BSA, and centrifuged 200g for 5 min to isolate floating mature adipocytes. Purified nuclei were fixed with 1% formaldehyde; lysed for 10 min in ChIP lysis buffer (1% SDS, 10 mM EDTA, 50 mM Tris-HCl, pH 8.0, proteinase inhibitors, 1 mM PMSF, 20 mM Na Butyrate) and sonicated for 30 sec ON/OFF for 10 min in a Bioruptor^®^Pico (Diagenode) into ~200 base-pair fragments. After sedimentation, the supernatant was diluted 10 times and chromatin incubated with anti-H3K27ac (Diagenode c15410174), anti-H3K4me3 (Diagenode c15410003), anti-H3K27me3 (Diagenode c15410069) or anti-H3K4me1 (Diagenode c15410037) antibodies, each at 2.5 μg/106 cells, for 2 h at 4°C. ChIP samples were washed, cross-links reversed, and DNA eluted for 2 h at 68°C. DNA was purified using phenol-chloroform isoamylalcohol and dissolved in H_2_O. Libraries were prepared using a Microplex kit (Diagenode) and sequenced on a NextSeq 500 or NovaSeq (Illumina).

ChIP-Seq reads for histone modifications were filtered with fastp, aligned to the genome with bowtie2, and deduplicated (**Supplementary Table 2**). Log ratio ChIP/Input tracks were generated using deepTools bigwigCompare from reads per genome coverage (RPGC) normalized tracks, created with bamCoverage. Profile plots were generated using deeptools computeMatrix and plotProfile.

PPARγ and MED1 ChIP-Seq were downloaded from Gene Expression Omnibus (GEO) accession GSE59703 (33). Reads from replicates were merged and aligned to hg38 using Bowtie2 (**Supplementary Table 2**). Peaks were detected using MACS3. ChIP/Input ratio tracks were generated using bamCompare in deepTools. PPARγ and MED1 ChIP/Input mean ratios were calculated for +/- 250 bp windows around the TSS using multiBigwigSummary from deepTools.

### 2.9 Alphafold predictions

Energy minimized structures of PEMT isoforms were predicted using Alphafold via ColabFold (34). Molecular graphics and analyses performed with UCSF ChimeraX (35).

### 2.10 Statistical analysis

Fisher’s exact test, implemented in ClusterProfiler (36), was used for overrepresentation analysis of genes in ontologies and pathways from MSigDb v2023.1 (37). For bioinformatic analyses, two-way ANOVA were conducted in R, followed by pairwise t-tests or Wilcoxon tests with Holmberg adjustment for multiple comparisons.

## 3 Results

### 3.1 ASC-derived adipocytes have differential beiging capacity

**Figure 1:**
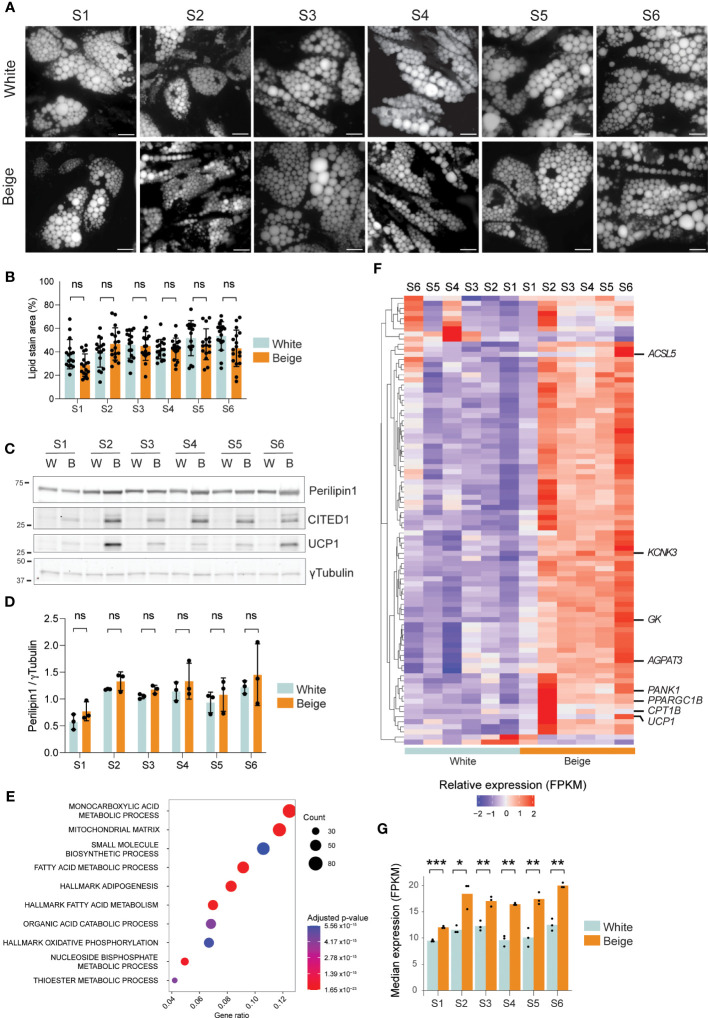
Model validation. **(A)** Bodipy staining of lipid droplets of day 15 differentiated white and beige adipocytes from 6 unrelated human subjects. Scale bar: 10µM. **(B)** Lipid droplet area per field quantified from **(A)** (ns non-significant; two-way ANOVA with Tukey’s multiple comparison; n>= 15 fields per condition from 3 independent experiments). **(C)** Western blot analysis of Perilipin1, CITED1, and UCP1 expression in day 15 differentiated white (W) and beige (B) adipocytes from 6 subjects. γTubulin is shown as a loading control. **(D)** Perilipin1 signals normalized to γTubulin, quantified from western blots (ns, non-significant; two-way ANOVA with Sidák’s multiple comparisons test; n=3). **(E)** Overrepresentation analysis of genes upregulated in beige vs white adipocytes from all donors (p < 0.05; RNA-seq) using GO and Hallmark gene sets from MSigDb (adjusted p-values < 1.2 x10^-6^). The gene ratio is the fraction of differentially expressed genes in each gene set. **(F)** Heatmap of relative gene expression (z-score transformed FPKM) for differentially expressed beige marker genes (p < 0.01 in any donor, eBayes method, limma package) from the BATLAS gene set (37). **(G)** Median gene expression of beige marker genes from the BATLAS gene set in day 15 white and beige adipocytes from 6 human subjects (*p < 0.05, **p < 0.01, ***p < 0.001; two-way ANOVA and t-tests with Holmberg adjustment).

To characterize the alternative splicing events that may specify the beige thermogenic phenotype of human adipocytes, we differentiated primary ASCs from 6 unrelated, normal-weight female subjects (S1-S6) into beige or white adipocytes, in the presence or absence of rosiglitazone, respectively. After 15 days, differentiation efficiency is similar in all 6 ASC line-derived adipocytes, based on lipid droplet accumulation (**Figures 1A, B**) and Perilipin1 expression (**Figures 1C, D**, **Supplementary Figure 1**). As expected, rosiglitazone treatment elicits the expression of the beige adipocyte markers CITED1 and UCP1 in all donors (**Figure 1C**), albeit to a different extent, confirming that efficient beige adipogenesis is achieved with all 6 ASC lines. Accordingly, transcriptome analysis shows that differentially expressed genes (DEGs) in beige vs white adipocytes from all subjects are significantly enriched for Gene Ontology (GO) terms “Mitochondrial matrix” (GO:0005759), “Monocarboxylic acid metabolic process” (GO:0032787) and “Fatty acid metabolic process” (GO:0006631) (**Figure 1E**, **Supplementary Table 4**). Further analysis of the thermogenic gene expression signature using BATLAS beige-curated gene list (38) clearly segregates white from beige differentiated adipocytes (**Figure 1F**), with the median expression of BATLAS beige markers being significantly increased in beige adipocytes (**Figure 1G**). Hence, PPARγ agonist treatment efficiently elicits a beige adipocyte transcriptional program in all 6 ASC lines.

### 3.2 Differential splicing in beige and white adipocytes gives rise to functionally distinct transcript isoforms

**Figure 2:**
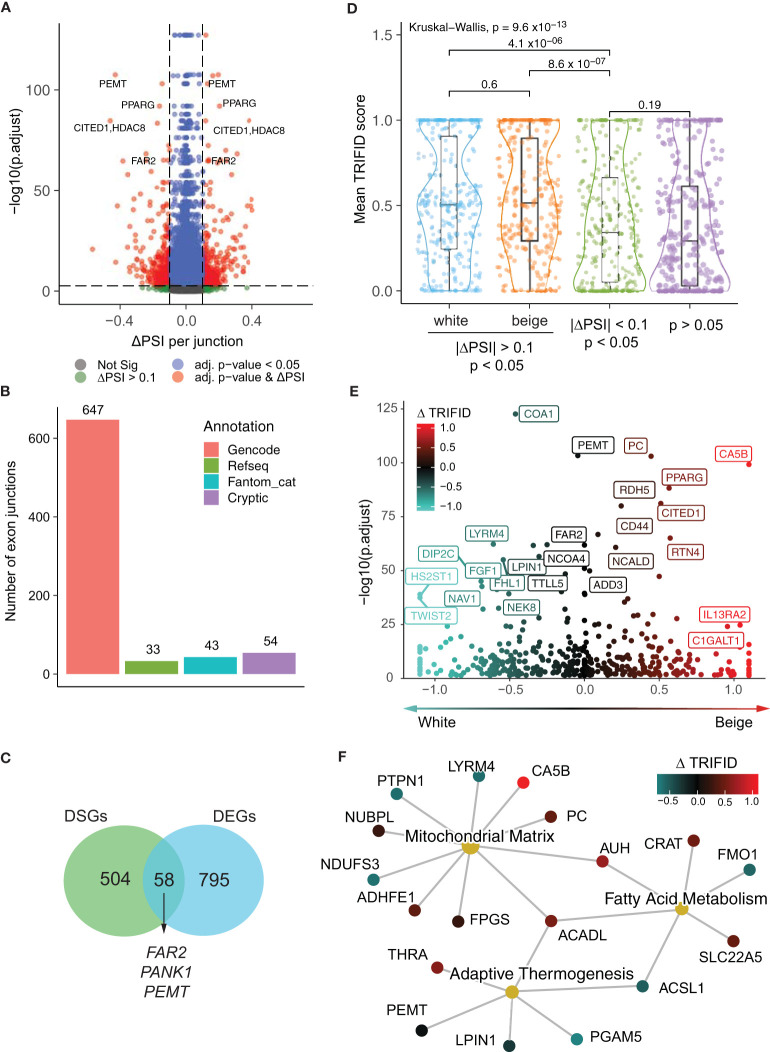
Functional classification of beige and white alternative isoforms. **(A)** Volcano plot of changes in exon-exon junction percentage spliced-in (PSI) between white and beige adipocytes and adjusted p-values per cluster from LeafCutter. **(B)** Annotation of exon junctions to transcript databases. **(C)** Venn diagram showing the overlap between differentially spliced genes (DSGs) and differentially expressed genes (DEGs) **(D)** Mean TRIFID score for exon junctions with white and beige enrichment (| ΔPSI> 0.1 & p < 0.05) compared to junctions with low differential enrichment (| **Δ**PSI< 0.1) from significant clusters (p < 0.05; n subsampled to 280), and non-significant junctions (p > 0.05; n subsampled to 280; Kruskal-Wallis test and t-tests with Holmberg adjustment). **(E)** Change in normalized TRIFID scores (Δ TRIFID) between white and beige isoforms (averaged per exon junction) plotted against LeafCutter ΔPSI adjusted p-value. **(F)** Network representation of genes pertaining to “Mitochondrial Matrix” (GO:0005759) “Fatty acid metabolism” (GO:0006631) and “Adaptive thermogenesis” (GO:1990845) GO terms, colored by Δ TRIFID score.

We next assessed differential splicing events in the white and beige adipocyte transcriptomes using LeafCutter, an annotation-free method that allows the identification and quantification of alternative splicing events by focusing on intron excision events, which are inferred from reads spanning exon–exon junctions (15). LeafCutter analysis identifies 777 exon junctions with robust differential usage between white and beige adipocytes in all 6 subjects (**Figure 2A**, **Supplementary Table 5**). Strikingly, genes with highly significant differences in intron excision, quantified by percent spliced-in (ΔPSI), include beiging markers (*CITED1*, *PANK1*), genes involved fatty acid and phospholipid remodeling (*FAR2, PEMT, PLD1, LPIN1*) or glucose metabolism (*PC*) as well as *PPARG* itself. Amongst the cell type specific exon junctions detected by LeafCutter, 723 could be annotated to known transcripts from either Gencode, RefSeq, or Fantom databases (**Figure 2B**). However, the remaining 54 cryptic exon junctions display canonical 5’ and 3’ motifs, and mostly result from differential 5´ splice site usage (**Supplementary Figures 2A-C**). Differential exon junctions detected in the beige vs white adipocyte transcriptomes map to 562 genes. Strikingly, only a minor fraction of these differentially spliced genes (DSGs) are also differentially expressed (**Figure 2C**), indicating that differential isoform expression constitutes an additional mechanism for gene expression regulation during beige adipocyte differentiation.

To assign functionality to differential exon junction usage, we mapped each junction to its matching transcripts and leveraged the TRIFID database to score protein functionality (18). TRIFID predicts protein functionality based on a number of transcript features including the presence of functional domains, cross-species conservation, transcript length and annotation database agreement. The TRIFID score (between 0 and 1) represents the likely functional relevance of protein isoforms. Both beige- and white-specific transcripts using spliced-in exon junctions have significantly higher average TRIFID functionality scores compared to the total transcriptome and to non-enriched exon-exon junctions from the same genes (**Figure 2D**), supporting a functional outcome for alternative isoform expression. Indeed, differential TRIFID score (ΔTRIFID) for DSGs predicts higher functionality for *PC, CA5B, CITED1, RTN4* and *PPARG* isoforms in beige adipocytes (**Figure 2E**, **Supplementary Table 6**), and genes related to “Adaptive thermogenesis”, “Fatty acid metabolism” and “Mitochondrial matrix” show either an increase or decrease in predicted isoform functionality in beige adipocytes (**Figure 2F**). Altogether, these results argue for a functionally relevant impact of alternative isoform expression on the regulation of beige vs white adipocyte functions.

### 3.3 Beige-specific isoforms arise from differential TSS usage

**Figure 3:**
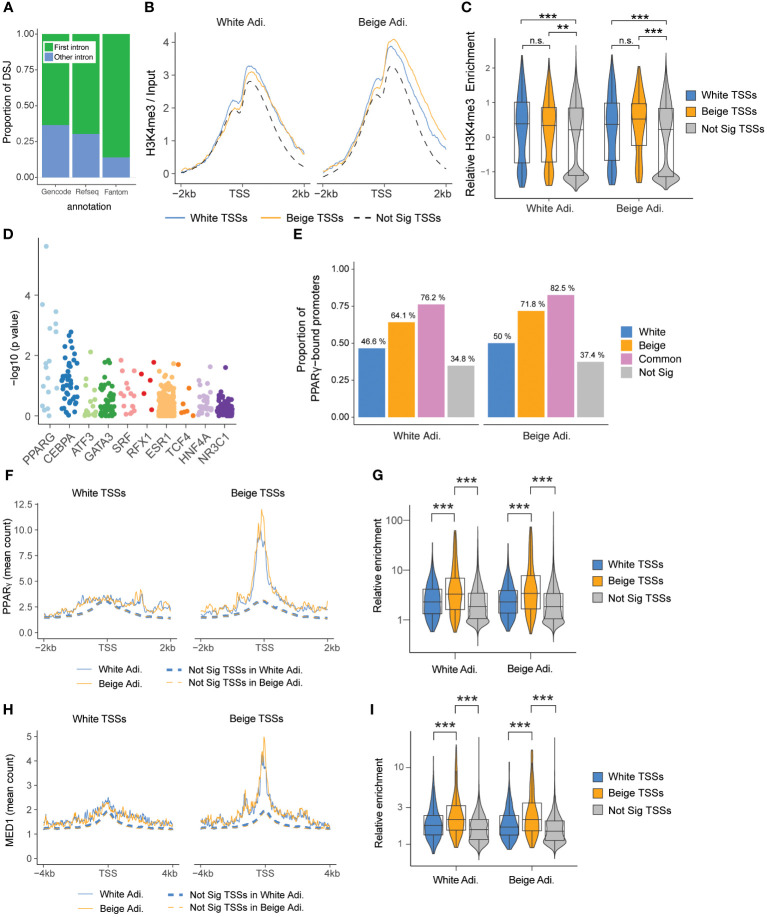
Splice variants arise from differential TSS usage. **(A)** Proportion of differentially spliced junctions (DSJs) mapping to the first intron of a transcript, arranged by annotation database. **(B)** Enrichment of H3K4me3 ChIP-Seq (log ratio of input) at differential TSSs in white and beige adipocytes. TSSs belonging to non-significant DEGs and non-significant DSGs are used as a control (n=23,529). **(C)** Quantification of H3K4me3 enrichment around the TSS (-1 kb/+2 kb), scaled within each condition (ns, non-significant, ** p < 0.01: ***p < 0.001; two-way ANOVA and Wilcoxon test with Holmberg adjustment). **(D)** Top 10 differentially enriched transcription factor binding sites at beige vs white TSSs (-2 kb/+0.5 kb) identified by Unibind. **(E)** Proportion of promoters (TSSs -2 kb/+0.5 kb) that intersect PPARγ ChIP-Seq peaks (beige promoters n=170; white promoters n=174) and promoters from non-significant DSGs and DEGs (n=12 257). Overlapping white and beige promoter regions are shown separately (n=63). **(F, H)** PPARγ and MED1 ChIP profiles around white (left panel) and beige (right panel) TSSs. **(G, I)** Quantification of PPARγ (TSS ± 250 bp) and MED1 (TSS ± 500 bp) ChIP enrichment around white, beige, and non-significant TSSs (***p < 0.0001; two-way ANOVA and Wilcoxon test with Holmberg adjustment).

To understand the mechanism driving beige-specific intron excision events, we first mapped these events along transcripts. We find a large majority of differential events occurring in the first intron of transcripts (**Figure 3A**), suggesting that DSGs between white and beige adipocytes may result from alternative TSS usage. This is further supported by the absence of consistent changes in expression of splicing regulators between white and beige adipocytes, and the minor overlap with splicing-driven alternative transcript expression described for adrenergic-induced adipocyte beiging (**Supplementary Figures 3**, **4**) (28). We thus examined the epigenetic state of white and beige specific TSSs in mature adipocytes by ChIP-seq. Enrichment profiles for the active histone marks H3K4me3 and H3K27ac at beige TSSs are similar in beige and white adipocytes (**Figures 3B, C**, **Supplementary Figures 5A, B**), indicating that beige TSSs are already in an active state in white adipocytes. In line, we find a similar bimodal H3K4me1 enrichment around white and beige TSSs, and no enrichment for the repressive histone mark H3K27me3 is detected at beige TSSs in white adipocytes (**Supplementary Figures 5C-G**). Hence, differential TSS usage in beige vs white adipocytes is not driven by a transition in chromatin state.

### 3.4 Beige-specific TSSs are bound by PPARγ in beige and white adipocytes

We reasoned that beige TSS activation might result from differential binding of transcription factors. Using Unibind’s binding site predictions (39), we find PPARγ and CEBPα transcription factor binding sites as the most differentially enriched in beige compared to white TSSs (**Figure 3D**). Analysis of PPARγ ChIP-Seq data in human white and beige adipocytes (33) confirms that a higher proportion of beige TSSs overlap PPARγ peaks compared to white TSSs and non-significantly enriched TSSs (**Figure 3E**). Accordingly, average PPARγ enrichment levels are higher at beige TSSs in both beige and white adipocytes (**Figures 3F, G**). Since cell type-specific enhancers are enriched for MED1 binding in beige vs white adipocytes (33), we examined MED1 binding in each TSS subclass (**Figure 3H**). Similar to PPARγ, MED1 signals are significantly enriched at beige vs white TSSs (**Figure 3I**). However, rosiglitazone treatment does not result in increased MED1 binding, indicating that promoter-enhancer contacts at beige TSSs are also established in white adipocytes (**Figures 3H, I**). Altogether, our results show that beige-specific TSSs are in an active chromatin state and bound by PPARγ in both white and beige adipocytes, implying that rosiglitazone activation of PPARγ triggers cell type-specific isoform expression in beige adipocytes.

### 3.5 Distinct PPARγ isoform balance between white and beige adipocytes

**Figure 4:**
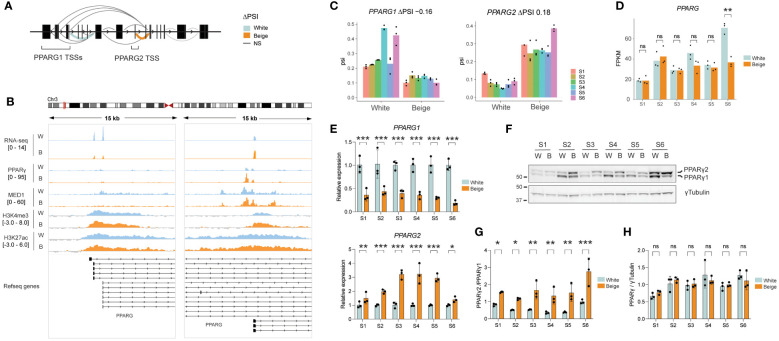
Beige specific isoform expression of PPARG. **(A)** Schematic representation of differential splicing pattern across the *PPARG* gene. **(B)** Integrative genomics viewer (IGV) tracks showing an overlay of S1-S6 RNA-seq reads on the forward strand, PPARγ and MED1 ChIP-seq over input ratios (33), and H3K4me3 and H3K27ac signals across *PPARG1* and *PPARG2* TSSs in beige vs white adipocytes. **(C)** ΔPSI on exon1-exon2 junction for *PPARG1* (left panel) and *PPARG2* (right panel) in white vs beige adipocytes derived from 6 ASC lines (p < 0.0001; LeafCutter). **(D)** Total *PPARG* expression level (FPKM) in white vs beige adipocytes (**p < 0.01, ns, non-significant; eBayes method, limma package; n=3) **(E)** Relative expression of *PPARG1* (upper panel) and *PPARG2* (lower panel) assessed by qPCR using isoform specific primers (*p < 0.05, **p < 0.01, ***p < 0.001; two-way ANOVA with Tukey’s multiple comparison; n=3). **(F)** Representative western blot and **(G, H)** quantification of PPARγ isoforms and total protein expression normalized to γTubulin (*p < 0.05, **p < 0.01, ***p < 0.001; two-way ANOVA with Sidák’s multiple comparisons test; n=3).

LeafCutter analysis reveals that white and beige adipocytes express distinct proportions of *PPARG* isoforms (see **Figure 2A**). Indeed, exon junction reads spanning *PPARG1* exon 1 and 2 are overrepresented in white adipocytes, while *PPARG2* first exons are most expressed in beige adipocytes (**Figures 4A-C**). In agreement with whole TSS analyses, *PPARG1* and *PPARG2* TSSs are similarly enriched with broad domains of H3K4me3 and H3K27ac, marking active promoters (**Figure 4B**). However, only the *PPARG2* TSS region shows an enrichment for PPARγ transcription factor binding and several MED1 peaks, suggestive of promoter-enhancer interaction (**Figure 4B**). Interestingly, *PPARG2* TSS activation does not result in a global increase in *PPARG* expression in beige adipocytes (**Figure 4D**), but rather in a switch in the proportion of *PPARG* isoforms as confirmed by isoform-specific RT-qPCR (**Figure 4E**). Importantly, we show that both isoforms of PPARγ are readily detected at the protein level (**Figure 4F**), and rosiglitazone treatment results in a shift in PPARγ isoform expression with PPARγ2 becoming the main isoform in all ASC lines (**Figure 4G**), while total PPARγ protein expression remains unchanged (**Figure 4H**). Thus, altered balance of PPARγ isoform expression in beige adipocytes may underlie the activation of beige-specific TSSs upon rosiglitazone treatment.

### 3.6 Beige cryptic PEMT isoform is efficiently translated

**Figure 5:**
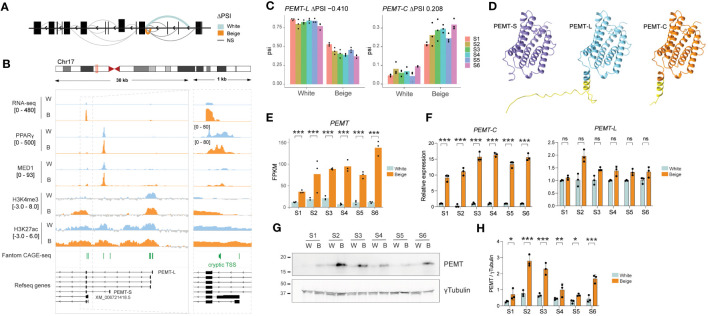
Beige-specific isoform expression of *PEMT.* **(A)** Schematic representation of differential splicing pattern across PEMT gene. **(B)** Integrative genomics viewer (IGV) tracks showing an overlay of S1-S6 RNA-seq reads on the reverse strand, PPARγ and MED1 ChIP-seq over input ratios (33), H3K4me3 and H3K27ac signals and Fantom CAGE-seq peaks across PEMT TSSs in white vs beige adipocytes. **(C)** ΔPSI on exon1-exon2 junction for *PEMT-L* (left panel) and *PEMT-C* (right panel) in beige vs white adipocytes derived from 6 ASC lines (p < 0.0001; LeafCutter). **(D)** Alphafold models for PEMT-S, PEMT-L and PEMT-C isoforms. The additional N-terminal amino acids in PEMT-L and PEMT-C are highlighted in yellow. **(E)** Total *PEMT* expression level (FPKM) in white vs beige adipocytes (***p < 0.0005, eBayes method, limma package, n = 3). **(F)** Relative expression of *PEMT-C* (left panel) and *PEMT-L* (right panel) assessed by qPCR using isoform specific primers (***p < 0.001, ns, non-significant, two-way ANOVA with Tukey’s multiple comparison, n = 3). **(G)** Representative western blot and **(H)** quantification of PEMT protein expression normalized to γTubulin (*p < 0.05, **p < 0.01, ***p < 0.001; two-way ANOVA with Sidák’s multiple comparisons test; n=3).

Amongst highly significant DSGs is *PEMT*, encoding the phosphatidylethanolamine methyltransferase (**Figures 5A–C**; see **Figure 2A**), an enzyme found at mitochondria-associated membranes and involved in the regulation of thermogenesis *in vivo* (40). *PEMT* encodes two major protein isoforms: short PEMT-S is liver-specific and highly active, while the long variant PEMT-L has an additional 37 N-terminal amino acids, reduced activity, and is expressed at low levels across a broad range of tissues (41) (**Figure 5D**; **Supplementary Figures 6** and **7**). Unlike most DSGs, *PEMT* expression is strongly induced in beige adipocytes (**Figure 5E**; see **Figure 2C**) and RNA-seq read alignment suggests this is driven by upregulation of a novel isoform (**Figures 5A, B**).

Indeed, LeafCutter identifies a switch from *PEMT-L* to a cryptic isoform of *PEMT (*hereafter referred to as *PEMT-C)* in beige adipocytes, with a first exon-exon junction matching a predicted transcript from RefSeq (XM_006721418.5) (**Figures 5A-C**), with a TRIFID functionality score similar to that of the *PEMT-L* isoform (see **Figure 2F**). However, no RNA-seq reads map over the first 250 bp of XM_006721418.5’s first intron, suggesting *PEMT-C* rather arises from a second downstream TSS, which is readily detected in Fantom robust CAGE-seq clusters (**Figure 5B**, right panel), and is a prominent *PEMT* TSS in differentiated adipocytes based on Fantom5 CAGE-Seq signal (**Supplementary Figure 8**). While *PEMT-C* promoter is similarly bound by PPARγ and MED1 in white and beige adipocytes, its upregulation in beige adipocytes correlates with an enrichment for the active promoter marks H3K4me3 and H3K27ac around its TSS (**Figure 5B**); this implies that the recruitment of chromatin modifiers underlies *PEMT-C* isoform expression in beige adipocytes.

Analysis of *PEMT* isoform expression using primers spanning isoform-specific exon junctions reveals that *PEMT* upregulation in beige adipocytes results from the induction of *PEMT-C* isoform only, while *PEMT-L* expression remains constant (**Figure 5F**). Importantly, only one PEMT isoform is detected at the protein level, with a molecular weight similar to that of the canonical PEMT-S isoform (**Figure 5F**). In agreement with RNA-seq and qPCR analysis, we find PEMT protein expression is significantly increased in beige adipocytes derived from all 6 ASC lines (**Figures 5G, H**). Thus, beige adipocytes express high levels of a novel PEMT isoform variant which is structurally close to the highly active PEMT-S variant (**Figure 5D**). Altogether, our results indicate that differential alternative TSS usage significantly impacts the beige adipocyte proteome.

## 4 Discussion

An increasing number of studies indicate that expression of alternative transcript isoforms plays an important role in adipose tissue physiology and pathophysiology (42). By analyzing differential gene and transcript expression in white and beige adipocytes differentiated from 6 human ASC lines, we uncover consistent switches in splicing patterns for more than 500 genes between the two lineages, likely resulting in the expression of functionally distinct protein isoforms.

Alternative splicing is a major driver of transcription heterogeneity, and splicing variations linked to age, sex, and ancestry contribute to phenotypic diversity (43). Our approach based on paired, parallel white and beige adipose differentiation from 6 unrelated subjects circumvents the issue of interindividual variability within the subpopulation. We identified splicing events using LeafCutter, a computational tool that estimates differential splicing directly from splice junction reads (15). While this approach has the major advantage of being annotation-independent, thus allowing cryptic event discovery, it also presents some limitations. Indeed, it cannot identify retained introns and relies on high read coverage across exon junctions, limiting splicing event identification for lowly expressed genes. However, the fact that only a fraction of DSGs are also differentially expressed suggests that variation in expression levels is not a strong bias in our dataset. In addition, interpretation of individual exon junction events is complicated by the relativization of PSI within clusters, which may link adjacent genes due to low coverage readthrough transcripts. However, linked genes are clearly identified in the output. Thus, by using an exon junction approach combined with stringent cut offs, we identify a set of robust, high-confidence splicing patterns characterizing white vs beige adipocytes in our system.

Recent evidence has highlighted a potential role for alternative splicing in the regulation of noradrenergic cAMP-mediated adipocyte beiging (25, 28). Indeed, sustained adrenergic stimulation elicits a global downregulation of splicing factors' gene expression in the pathological context of pheochromocytoma (28). Interestingly, such transcriptional remodeling of the splicing machinery was not observed upon rosiglitazone-induced beiging, a discrepancy that likely results from inherently different transcriptomic responses elicited by PPARγ- and cAMP-induced adipocyte browning (44). Instead, we find that differential isoform expression upon rosiglitazone-induced beiging mostly results from alternative TSS usage, rather than from alternative splicing of downstream exons. Nonetheless, both studies point to differential isoform expression as an important regulatory mechanism for thermogenic adipocyte specification and activation.

Rosiglitazone is a PPARγ agonist, and it is therefore not unexpected to find enriched PPARγ binding at beige-specific TSSs. Sequencing of nascent RNA in rosiglitazone-treated 3T3-L1 adipocytes highlights a redistribution of transcription towards PPARγ-driven enhancers (45). Intriguingly, rosiglitazone treatment also triggers gene downregulation through decreased coactivator binding at sites devoid of PPARγ, a mechanism which could account for the absence of overall gene upregulation upon PPARγ-dependent TSS activation, as we observe for *PPARG* itself. The functional significance of the switch in PPARγ protein isoform expression we observe in human beige adipocytes is supported by a recent study showing the differential effect of Pparγ1 and Pparγ2 deficiency on weight gain and thermogenic capacity (46). Indeed, mice with selective deficiency of PPARγ1 maintain body temperature better than PPARγ2-deficient mice and are protected against rosiglitazone induced weight gain. These results and ours point to PPARγ2 as the main PPARγ isoform driving adipocyte thermogenic response to rosiglitazone.

We find that PPARγ agonist-induced beiging triggers a consistent and strong upregulation of a new PEMT isoform. PEMT is most active in the liver where it is expressed as a short isoform, while its activity is thought to be only marginal in other tissues where the longer variant is expressed. PEMT-deficient mice are cold intolerant, a phenotype which has been linked to both compromised hepatic glucose production (40) and to lack of UCP1 expression in the brown adipose tissue (47). While the effect of PEMT deficiency in white adipose tissue has not been investigated, *in vitro* studies suggest that PEMT upregulation is required for efficient adipogenic differentiation (48). Our data together with public datasets identifies an adipose-specific TSS for PEMT, leading to the expression of a cryptic isoform which only differs by 16 additional amino acids from the highly active, liver specific PEMT-S. While the specific activity of this isoform needs to be examined, its strong upregulation in beige adipocytes is likely to alter the phosphatidylcholine to phosphatidylethanolamine ratio, which may modulate mitochondrial dynamics and function (49). Interestingly, the PEMT gene contains a splice junction quantitative trait loci (sQTL) which is associated with triglyceride levels and waist-to-hip ratio in genome-wide association studies (50). The same study identified numerous sQTLs associated with cardiometabolic traits in subcutaneous adipose tissue, supporting the contribution of alternative isoform usage to metabolic health. Further studies involving single-nuclei, isoform-level analysis of transcript expression in human adipose tissue will be needed to further establish the physiological relevance of beige-specific transcript expression.

## Data availability statement

The datasets presented in this study can be found in online repositories. The names of the repository/repositories and accession number(s) can be found below: https://www.ncbi.nlm.nih.gov/geo/, GSE256262 https://www.ncbi.nlm.nih.gov/geo/, GSE256260 https://www.ncbi.nlm.nih.gov/geo/, GSE256261. The link to the code for data processing and analysis can be found here: https://github.com/sarahhp/splicing_thermogenesis.

## Ethics statement

The studies involving humans were approved by Regional Committee for Research Ethics for Southern Norway (REK). The studies were conducted in accordance with the local legislation and institutional requirements. The participants provided their written informed consent to participate in this study.

## Author contributions

SHP: Formal analysis, Investigation, Validation, Visualization, Writing – original draft, Writing – review & editing, Data curation. MA: Writing – review & editing, Data curation, Formal analysis. PC: Funding acquisition, Writing – review & editing. NB: Conceptualization, Funding acquisition, Investigation, Project administration, Supervision, Validation, Visualization, Writing – original draft, Writing – review & editing.
