# Supplementary figures and images for: Alternative isoform expression of key thermogenic genes in human beige adipocytes
Source: Front Endocrinol (Lausanne). 2024 May 24;15:1395750. doi: 10.3389/fendo.2024.1395750 (PMC11163967; doi:10.3389/fendo.2024.1395750)

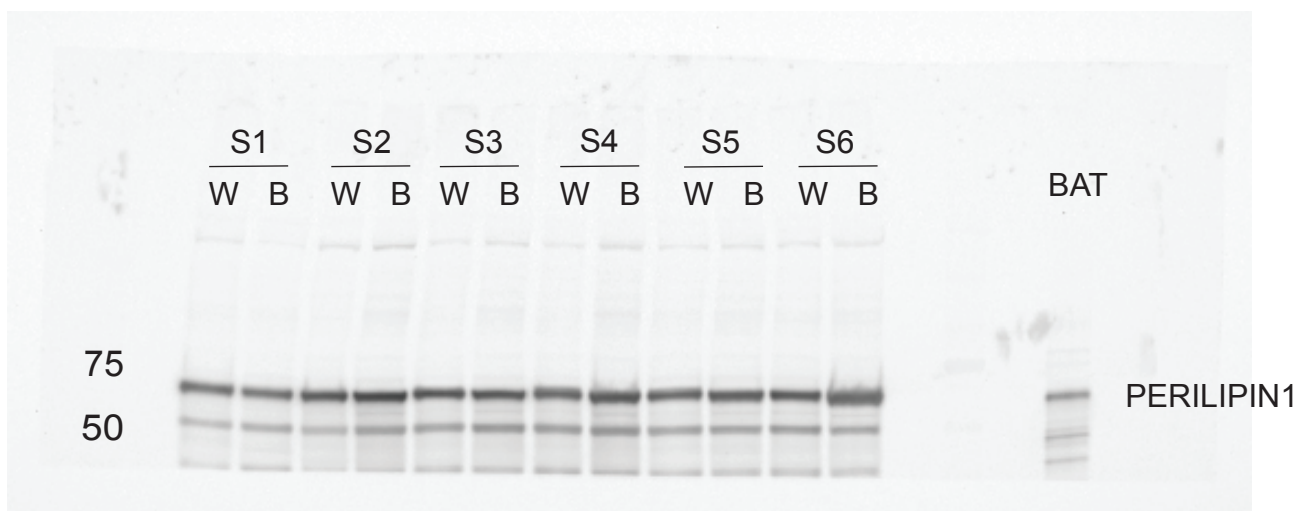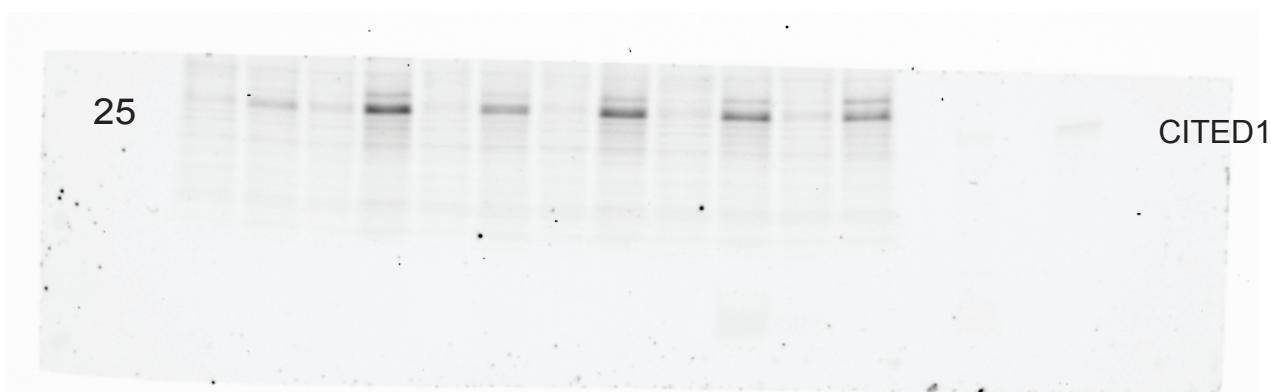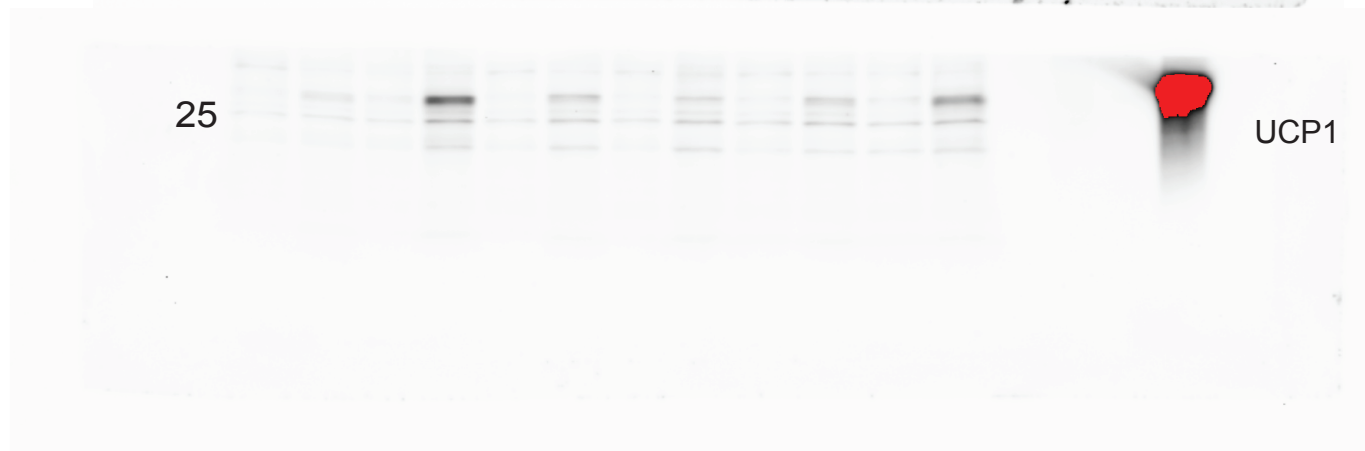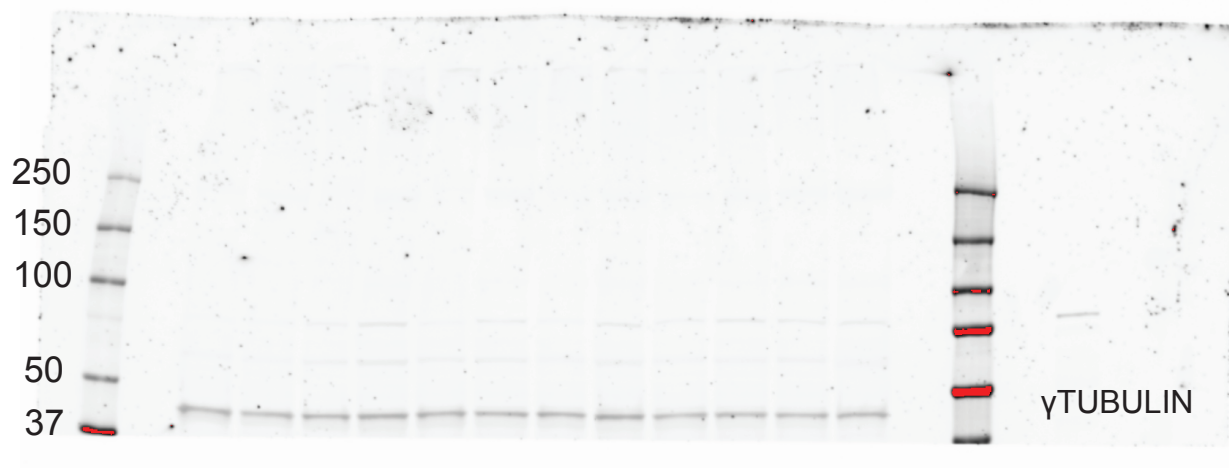

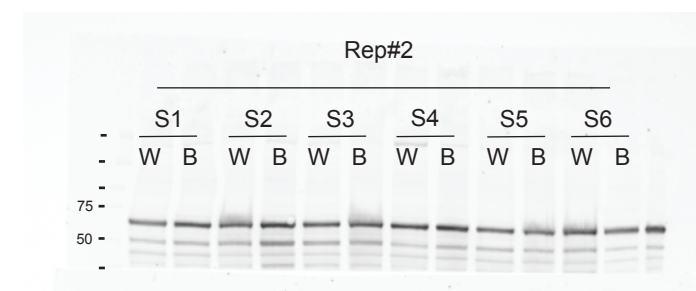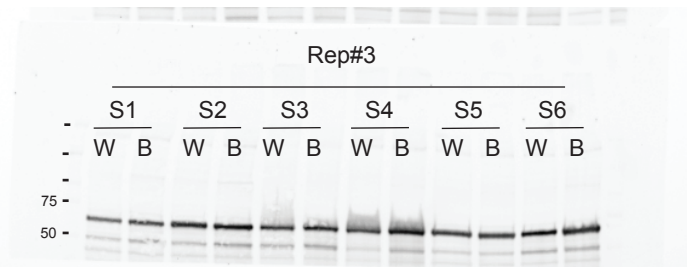

PERILIPIN1

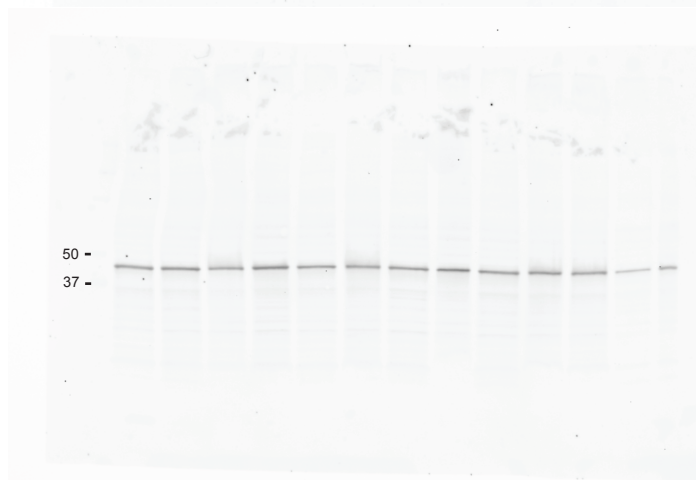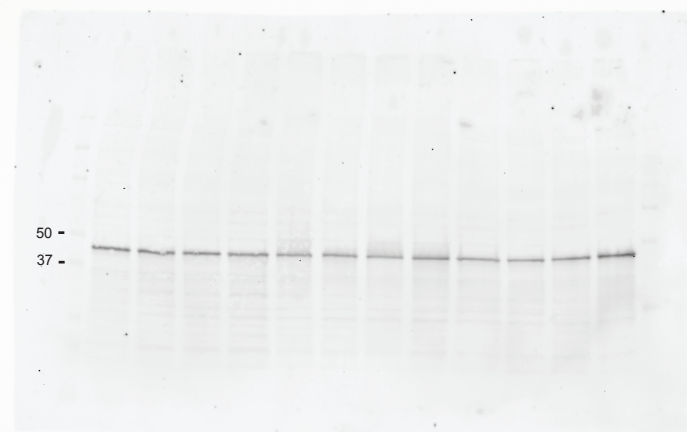

γTUBULIN

Supplementary data 2

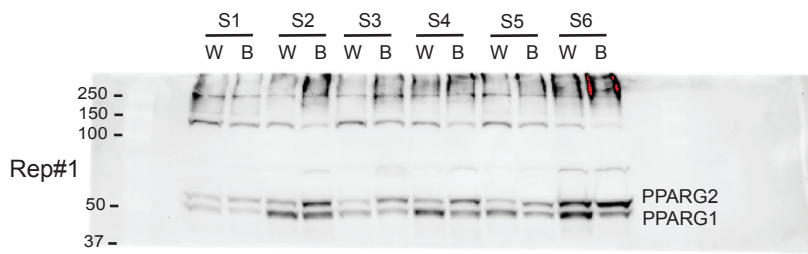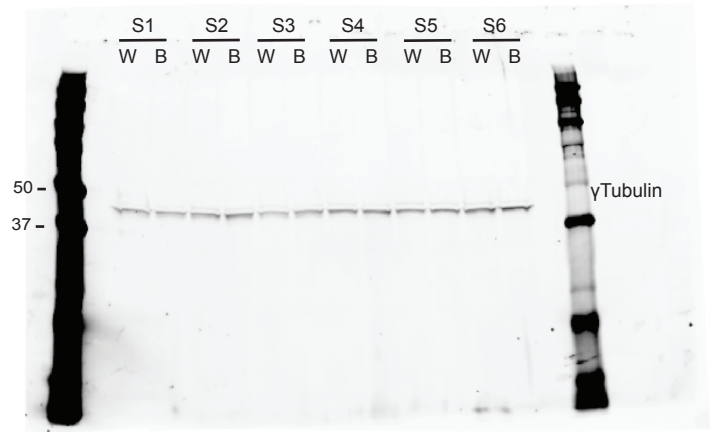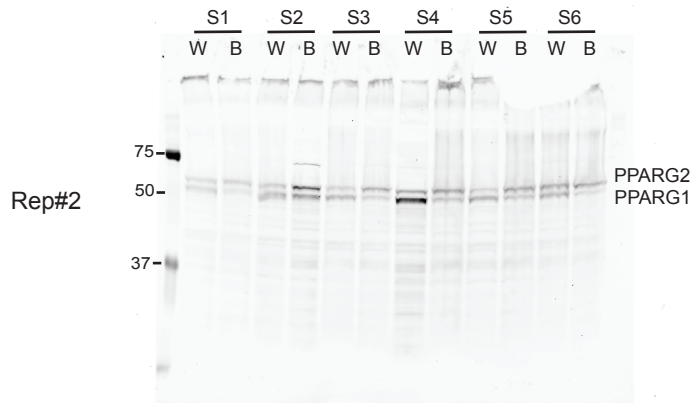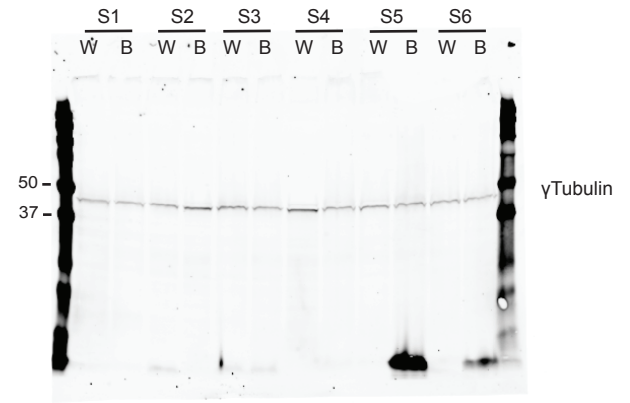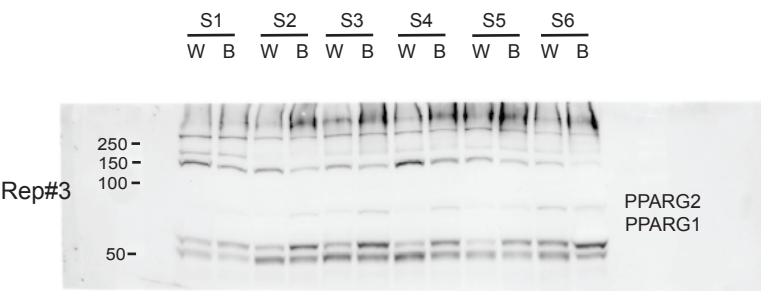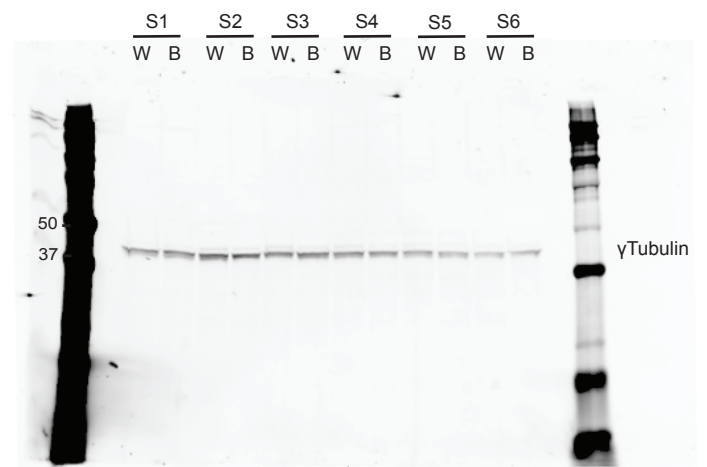

Supplementary data 3

Rep#1

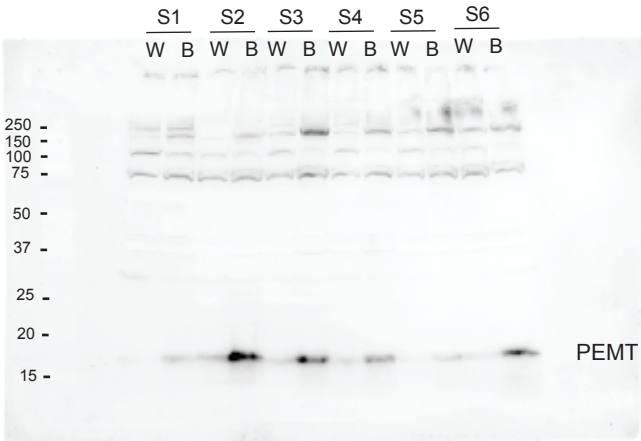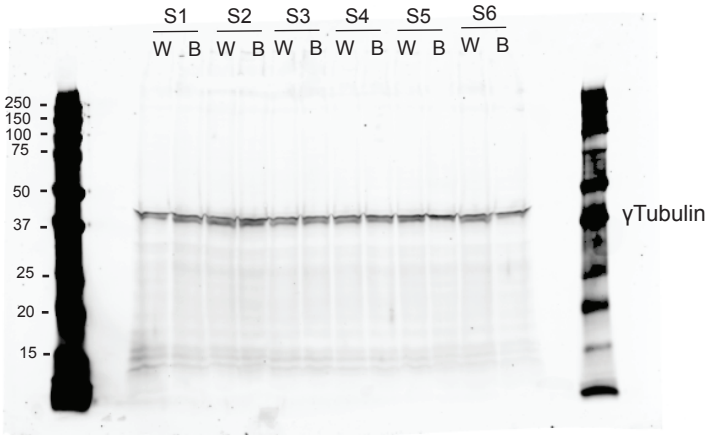

Rep#2

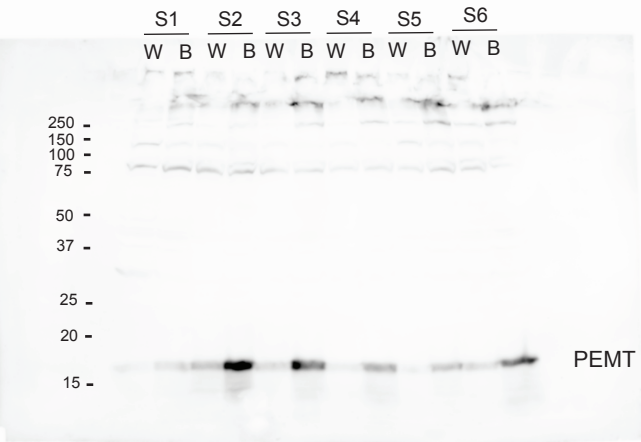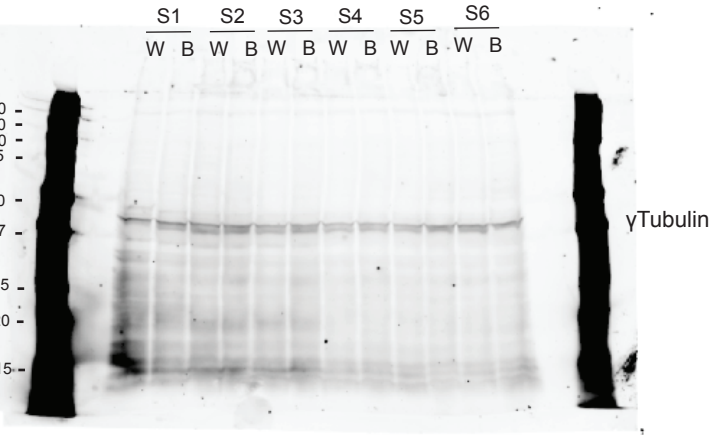

Rep#3

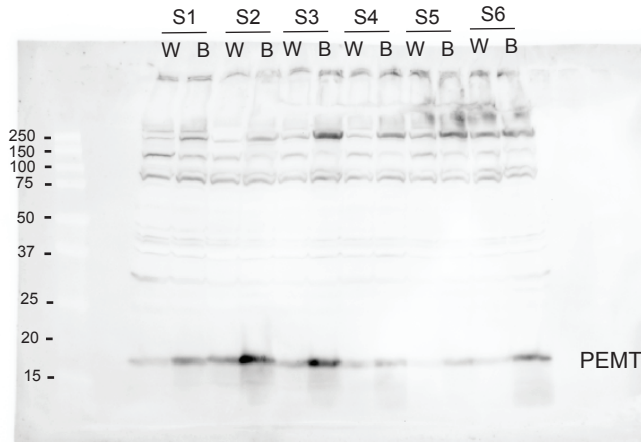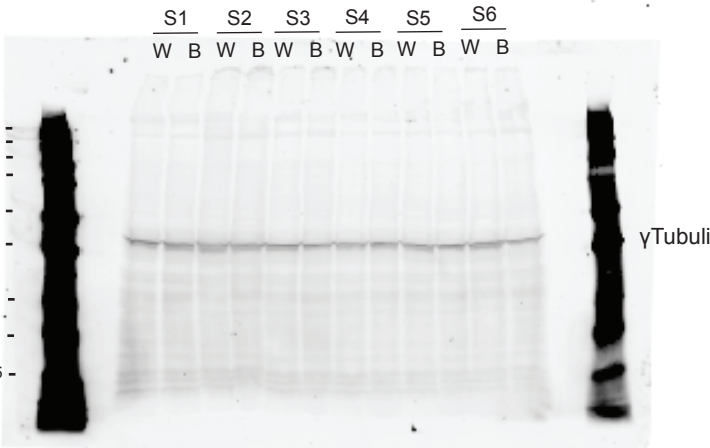

Supplementary data 4

Supplement: Supplementary file 1 [file DataSheet_1.pdf]
