# Supplementary material for: Alternative isoform expression of key thermogenic genes in human beige adipocytes
Source: Front Endocrinol (Lausanne). 2024 May 24;15:1395750. doi: 10.3389/fendo.2024.1395750 (PMC11163967; doi:10.3389/fendo.2024.1395750)
Supplement: Supplementary file 2 [file DataSheet_2.pdf]

frontiers

Supplementary Material

Alternative isoform expression of key thermogenic genes in human beige adipocytes

Sarah Hazell Pickering, Mohamed Abdelhalim, Philippe Collas, Nolwenn Briand*

*** Correspondence:** Corresponding Author: nolwenn.briand@medisin.uio.no

1 Supplementary Data

Supplementary Data 1. Uncropped membranes for Figure 1C
Supplementary Data 2. Uncropped membranes for Supplementary Figure 1
Supplementary Data 3. Uncropped membranes for Figure 4F and Supplementary Figure 9
Supplementary Data 4. Uncropped membranes for Figure 5F and Supplementary Figure 10

2 Supplementary Figures and Tables

Supplementary Figure 1. Perilipin1 expression in white and beige adipocytes
Supplementary Figure 2. Classification of cryptic exon junctions
Supplementary Figure 3. Heatmap of splicing factors gene expression
Supplementary Figure 4. Rosiglitazone- vs pheochromocytoma-induced transcripts overlap.
Supplementary Figure 5. Enrichment profiles and heatmaps for histone marks
Supplementary Figure 6. Protein sequence alignments of PEMT isoforms
Supplementary Figure 7. Expression of PEMT isoforms
Supplementary Figure 8. Expression of PEMT TSSs from CAGE-seq
Supplementary Figure 9. PPARγ isoform expression in white and beige adipocytes
Supplementary Figure 10. PEMT expression in white and beige adipocytes

Supplementary Table 1. Subject characteristics
Supplementary Table 2. Bioinformatics tools
Supplementary Table 3. qPCR primer sequences
Supplementary Table 4. Differential gene expression from limma eBayes tests per subject (.xlsx)
Supplementary Table 5. Exon junctions identified by Leafcutter (.xlsx)
Supplementary Table 6. Average and ΔTRIFID scores for each exon junction (.xlsx)

Supplementary Material

1.1 Supplementary Figures

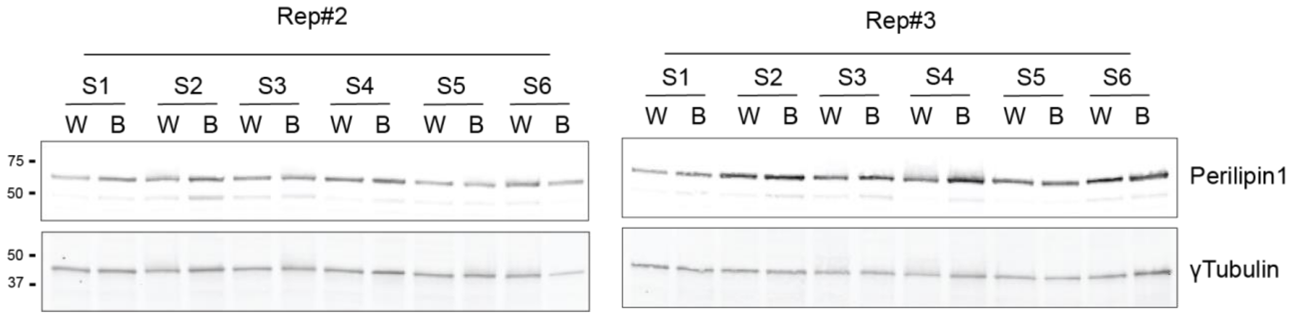

Supplementary Figure 1. Western blot analysis of Perilipin1 expression in day 15 differentiated white (W) and beige (B) adipocytes from 6 subjects. γTubulin is shown as a loading control.

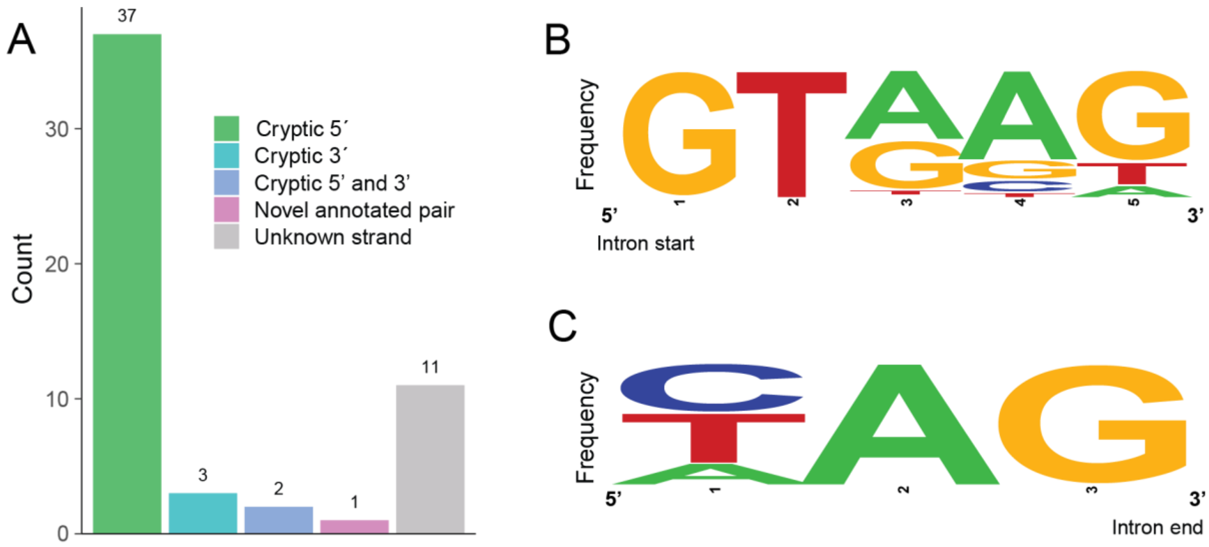

Supplementary Figure 2. Classification of cryptic exon junctions **(A)** Classification **(B)** Cryptic 5' motifs **(C)** Cryptic 3' motifs

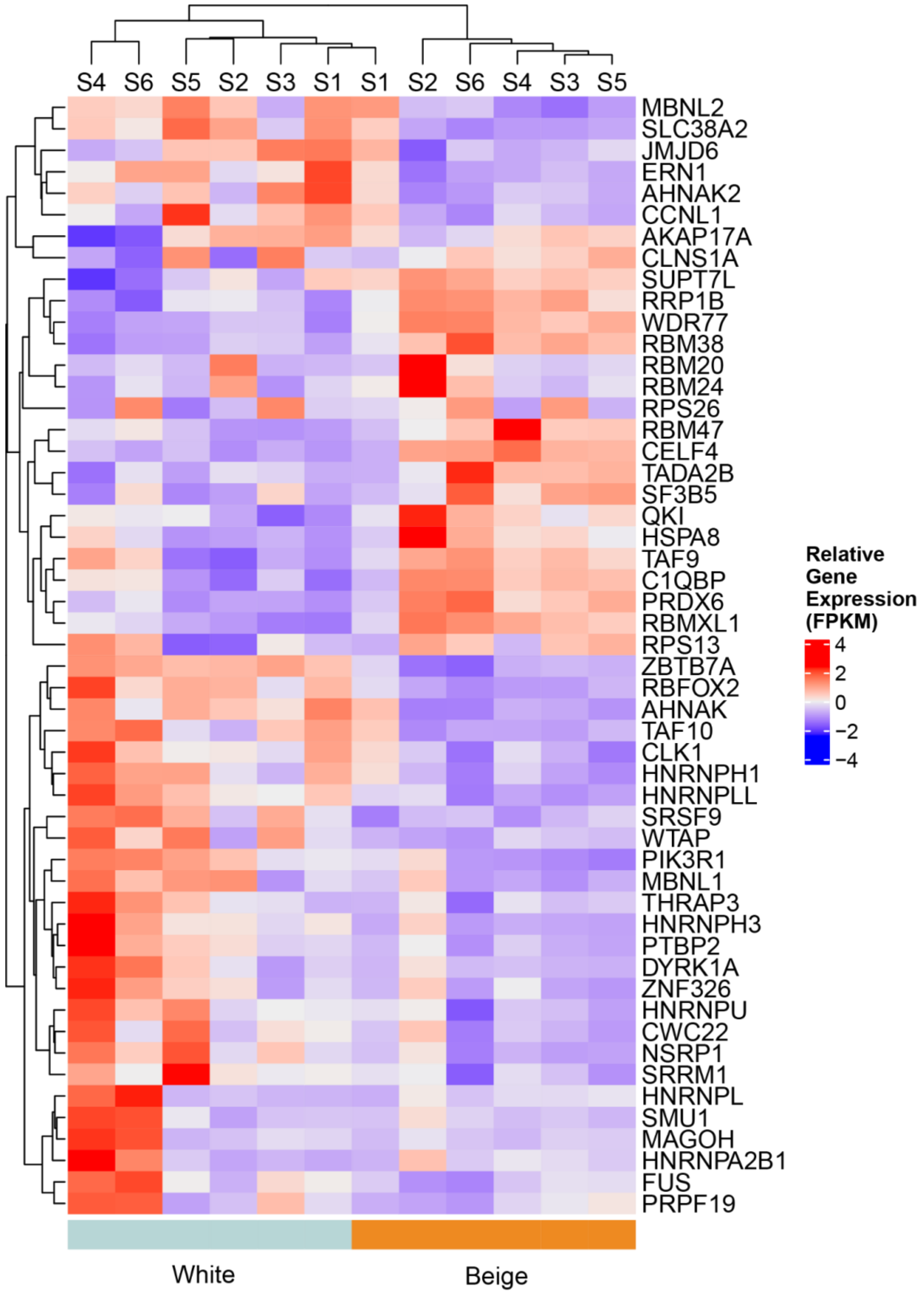

Supplementary Figure 3. Heatmap of splicing factors gene expression. Expression of splicing factor genes from GO term Regulation of RNA splicing (GO:0043484), with differential expression in at least one subject ($p < 0.01$)

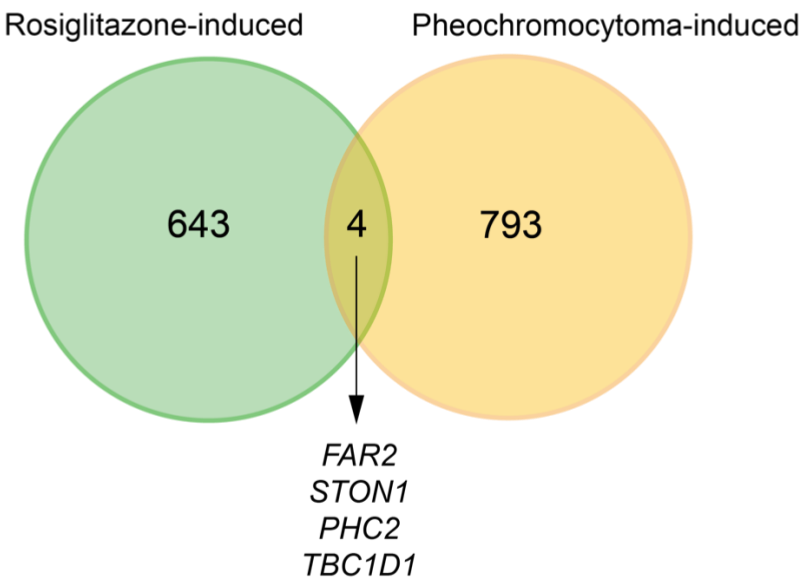

Supplementary Figure 4. Rosiglitazone- vs pheochromocytoma-induced transcripts overlap. Differentially used exon-exon junctions identified with LeafCutter in rosiglitazone-treated adipocytes, overlapped with differentially expressed transcripts in adipose tissue from pheochromocytoma patients (Castella *et al.* 2023). Only exon-exon junctions with a corresponding ensembl transcript were compared.

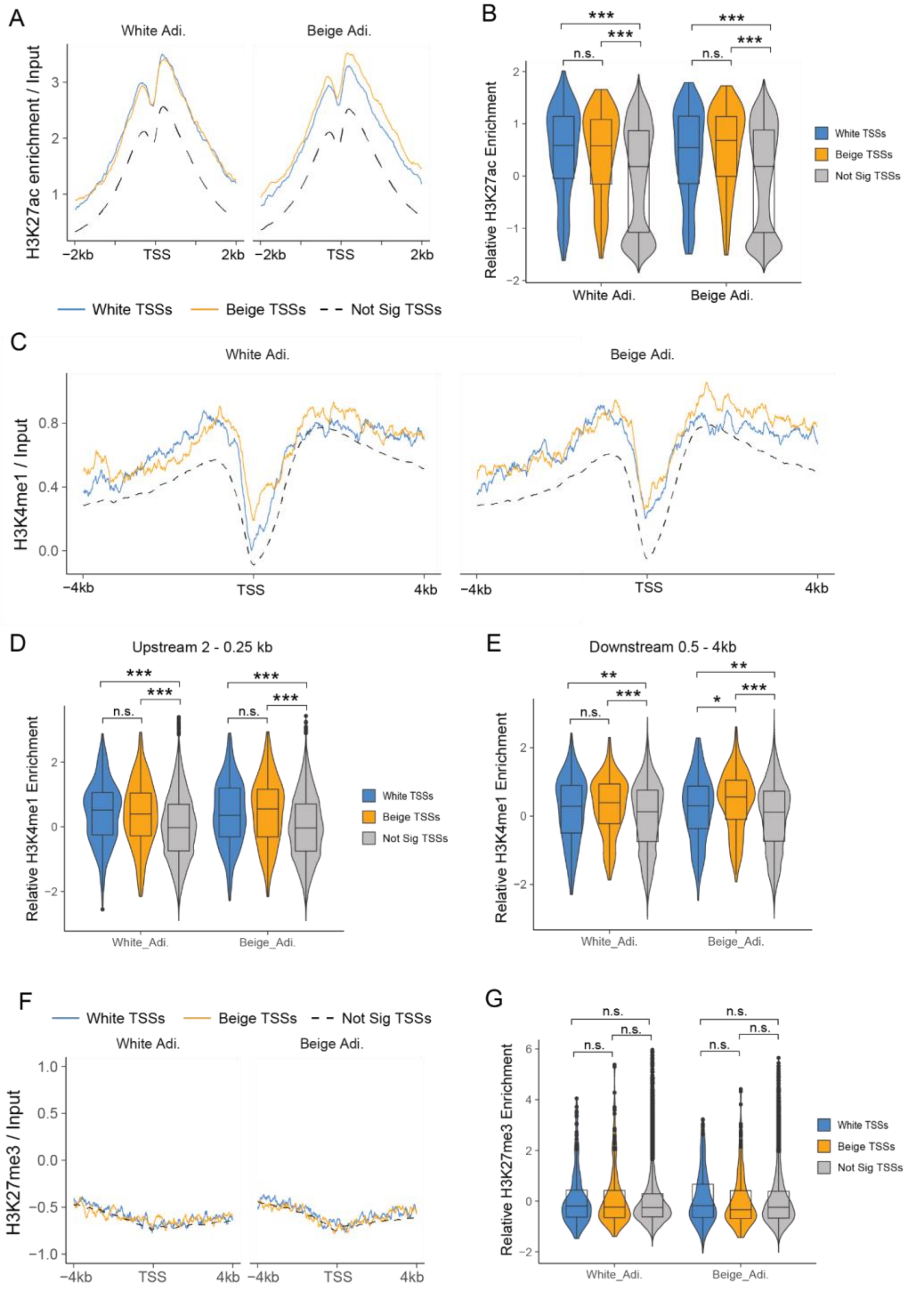

Supplementary Figure 5. Enrichment profile for histone marks at TSSs. (**A**,**C**,**F**) Enrichment of ChIP-Seq (log ratio of input) at differential TSSs in white and beige adipocytes and (**B**,**D**,**E**,**G**) quantification of ChIP-Seq enrichment around the TSS. **(A)** H3K27ac profile and **(B)** quantification +/- 1 kb around TSS. **(C)** H3K4me1 profile, **(D)** quantification upstream of the TSS -2 kb to -250 bp and **(E)** downstream of the TSS +500 bp to +4 kb. **(F)** H3K27me3 profile and **(G)** quantification +/- 2 kb around TSS. TSSs belonging to non-significant DEGs and non-significant DSGs are used as a control (n=26,636) (*p < 0.05, **p < 0.01, ***p < 0.001, two-way ANOVA and Wilcoxon test with Holmberg adjustment).

```
PEMT-C|crypticPEMT      --------------------------MFPGLPVCS-------QRADFCVMTRLLGYVDPL
XP_006721481.3          MTQLIRFQLFIFPFMRLHCSNLCRTWMFPGLPVCS-------QRADFCVMTRLLGYVDPL
PEMT-L|Q9UBM1-2         ------------MKRSGNPGAEVTNSSVAGPDCCGGLGNIDFQRADFCVMTRLLGYVDPL
PEMT-S|Q9UBM1           -------------------------------------------------MTRLLGYVDPL
                                                                         ***********

PEMT-C|crypticPEMT      DPSFVAAVITITFNPLYWNVVARWEHKTRKLSRAFGSPYLACYSLSVTILLLNFLRSHCF
XP_006721481.3          DPSFVAAVITITFNPLYWNVVARWEHKTRKLSRAFGSPYLACYSLSVTILLLNFLRSHCF
PEMT-L|Q9UBM1-2         DPSFVAAVITITFNPLYWNVVARWEHKTRKLSRAFGSPYLACYSLSVTILLLNFLRSHCF
PEMT-S|Q9UBM1           DPSFVAAVITITFNPLYWNVVARWEHKTRKLSRAFGSPYLACYSLSVTILLLNFLRSHCF
                        ************************************************************

PEMT-C|crypticPEMT      TQAMLSQPRMESLDTPAAYSLGLALLGLGVVLVLSSFFALGFAGTFLGDYFGILKEARVT
XP_006721481.3          TQAMLSQPRMESLDTPAAYSLGLALLGLGVVLVLSSFFALGFAGTFLGDYFGILKEARVT
PEMT-L|Q9UBM1-2         TQAMLSQPRMESLDTPAAYSLGLALLGLGVVLVLSSFFALGFAGTFLGDYFGILKEARVT
PEMT-S|Q9UBM1           TQAMLSQPRMESLDTPAAYSLGLALLGLGVVLVLSSFFALGFAGTFLGDYFGILKEARVT
                        ************************************************************

PEMT-C|crypticPEMT      VFPFNILDNPMYWGSTANYLGWAIMHASPTGLLLTVLVALTYIVALLYEEPFTAEIYRQK
XP_006721481.3          VFPFNILDNPMYWGSTANYLGWAIMHASPTGLLLTVLVALTYIVALLYEEPFTAEIYRQK
PEMT-L|Q9UBM1-2         VFPFNILDNPMYWGSTANYLGWAIMHASPTGLLLTVLVALTYIVALLYEEPFTAEIYRQK
PEMT-S|Q9UBM1           VFPFNILDNPMYWGSTANYLGWAIMHASPTGLLLTVLVALTYIVALLYEEPFTAEIYRQK
                        ************************************************************

PEMT-C|crypticPEMT      ASGSHKRS
XP_006721481.3          ASGSHKRS
PEMT-L|Q9UBM1-2         ASGSHKRS
PEMT-S|Q9UBM1           ASGSHKRS
                        ********
```

Supplementary Figure 6. Protein sequence alignments of PEMT isoforms. * indicates positions which have a single, fully conserved residue. XP_006721481.3 is the protein sequence corresponding to RefSeq isoform XM_006721418.5.

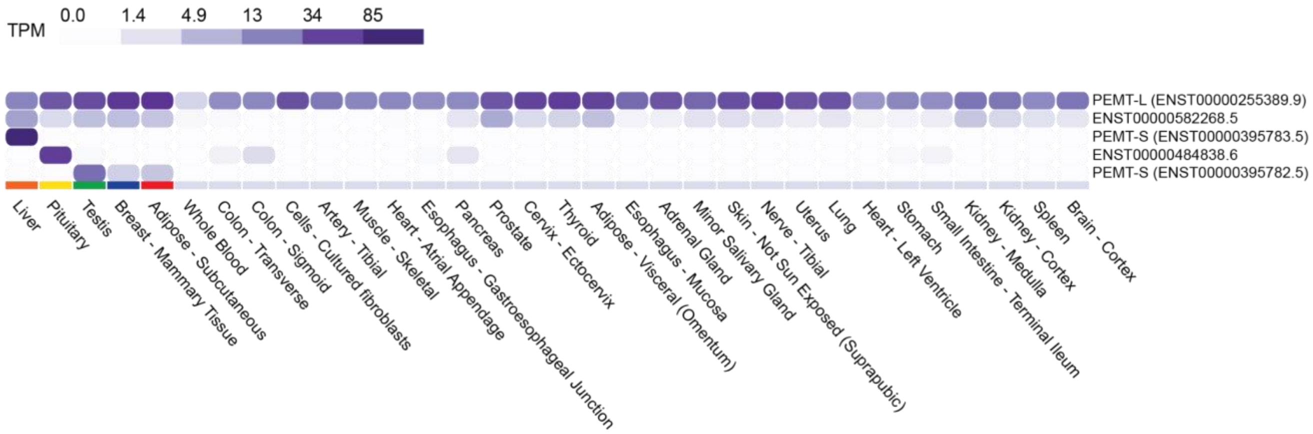

Supplementary Figure 7. Expression of PEMT isoforms. Expression of top PEMT transcript isoforms across human tissues. GTEx Analysis Release V8 includes only GENCODE isoforms.

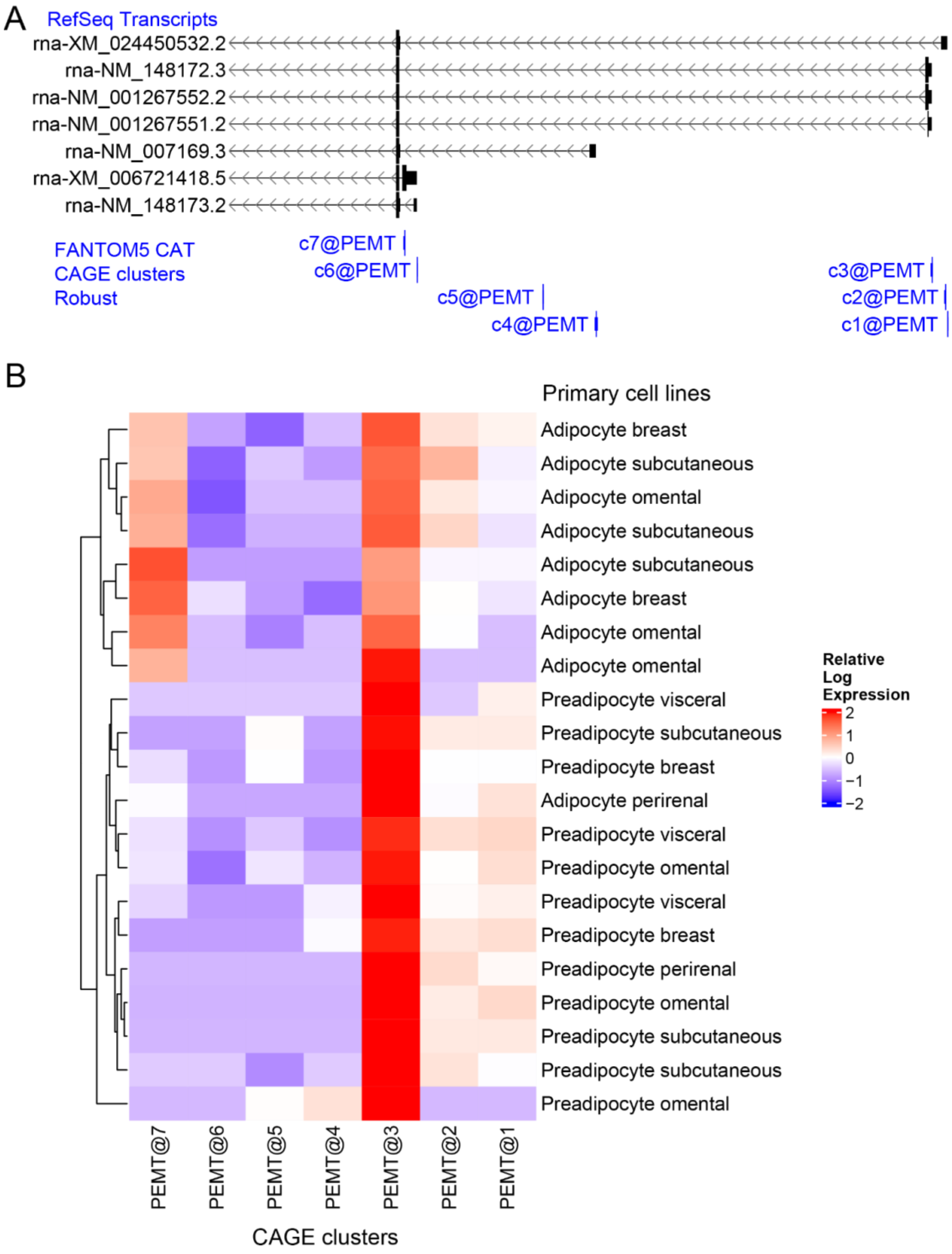

Supplementary Figure 8. Expression of PEMT TSSs from CAGE-seq. A Genome browser view of PEMT TSSs based on FANTOM5 CAGE clusters (cage associated transcriptome, robust set) (Hon et al. 2017) and associated RefSeq transcripts. **B** Fantom Robust CAGE reads (Relative Log Counts) for PEMT TSSs in adipocytes and adipose precursor samples from primary cell lines FANTOM5.

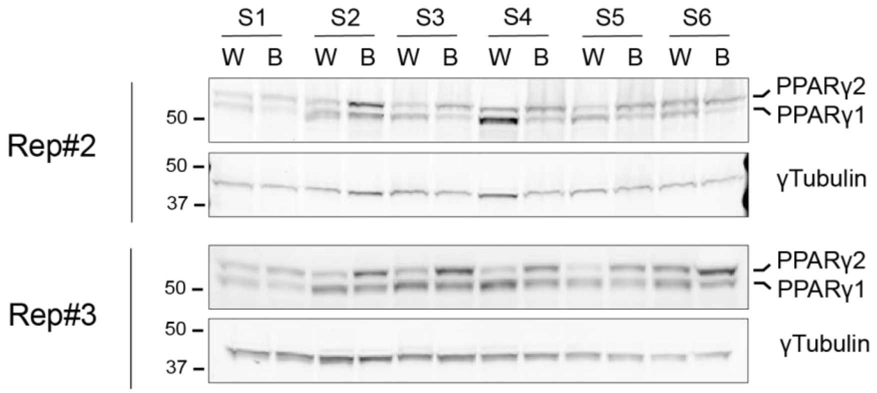

Supplementary Figure 9. PPARγ isoform expression in white and beige adipocytes. Western blot analysis of PPARγ expression in day 15 differentiated white (W) and beige (B) adipocytes from 6 subjects. γTubulin is shown as a loading control.

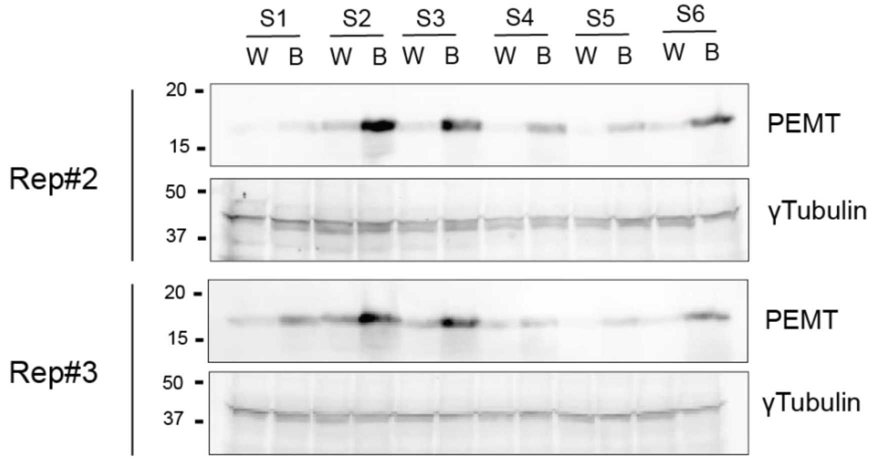

Supplementary Figure 10. PEMT expression in white and beige adipocytes. Western blot analysis of PEMT expression in D15 differentiated white (W) and beige (B) adipocytes from 6 subjects. γTubulin is shown as a loading control.

1.2 Supplementary Tables

Subject	Age (year)	BMI (kg/m²)
1	39	27,14
2	44	24,28
3	28	23,57
4	50	27,44
5	33	29,02
6	45	20,90

Supplementary Table 1. Subject characteristics

Tool	Dataset	Version	Reference
fastp	RNA-Seq, ChIP-Seq	0.20.1	Shifu Chen, Yanqing Zhou, Yaru Chen, Jia Gu, fastp: an ultra-fast all-in-one FASTQ preprocessor, *Bioinformatics*, Volume 34, Issue 17, September 2018, Pages i884–i890,
hisat2	RNA-Seq	2.1.0	Kim D, Paggi JM, Park C, Bennett C, Salzberg SL. Graph-based genome alignment and genotyping with HISAT2 and HISAT-genotype. Nat Biotechnol 2019 378. 2019 Aug 2;37(8):907–15.
featureCounts	RNA-Seq	2.0.1	Liao Y, Smyth GK, Shi W. featureCounts: an efficient general purpose program for assigning sequence reads to genomic features. Bioinformatics. 2014 Apr 1;30(7):923–30.
R	RNA-Seq, ChIP-Seq, GSE59703	4.2.2	
edgeR	RNA-Seq	3.40.2	Chen Y, Lun AAT, Smyth GK (2016). "From reads to genes to pathways: differential expression analysis of RNA-Seq experiments using Rsubread and the edgeR quasi-likelihood pipeline." *F1000Research*, **5**, 1438.
limma	RNA-Seq	3.54.2	Law CW, Chen Y, Shi W, Smyth GK. Voom: Precision weights unlock linear model analysis tools for RNA-seq read counts. Genome Biol. 2014 Feb 3;15(2):1–17.
clusterProfiler	RNA-Seq	4.6.2	Wu T, Hu E, Xu S, Chen M, Guo P, Dai Z, et al. clusterProfiler 4.0: A universal enrichment tool for interpreting omics data. Innovation. 2021 Aug 28;2(3).
STAR	RNA-Seq	2.7.10b	Dobin A, Davis CA, Schlesinger F, Drenkow J, Zaleski C, Jha S, et al. STAR: ultrafast universal RNA-seq aligner. Bioinformatics. 2013 Jan;29(1):15–21.<br>ENCODE options from manual:<br>https://github.com/alexdobin/STAR/blob/master/doc/STARmanual.pdf<br>--outFilterType BySJout --outFilterMultimapNmax 20<br>--alignSJoverhangMin 8<br>--alignSJDBoverhangMin 1<br>--outFilterMismatchNmax 999<br>--outFilterMismatchNoverReadLmax 0.04<br>--alignIntronMin 20<br>--alignIntronMax 1000000<br>--alignMatesGapMax 1000000
regtools	RNA-Seq	1.0.0	Cotto, K.C., Feng, YY., Ramu, A. *et al.* Integrated analysis of genomic and transcriptomic data for the discovery of splice-associated variants in cancer. *Nat Commun* **14**, 1589 (2023).

LeafCutter	RNA-Seq	0.2.9	Li YI, Knowles DA, Humphrey J, Barbeira AN, Dickinson SP, Im HK, et al. Annotation-free quantification of RNA splicing using LeafCutter. Nat Genet 2017 501. 2017 Dec 11;50(1):151–8. https://github.com/davidaknowles/leafcutter
deeptools	ChIP-Seq	3.4.3	Ramírez F, Dündar F, Diehl S, Grüning BA, Manke T. deepTools: a flexible platform for exploring deep sequencing data. Nucl Acids Res. 2014;42:W187–W91.
deeptools	GSE59703	3.5.3	
bowtie2	ChIP-Seq	2.3.5	Langmead B, Salzberg SL. Fast gapped-read alignment with Bowtie 2. Nat Methods. 2012;9:357–9
bowtie2	GSE59703	2.4.5	
macs2	ChIP-Seq	2.2.7.1	Zhang, Y.; Liu, T.; Meyer, C.A.; Eeckhoute, J.; Johnson, D.S.; Bernstein, B.E.; Nussbaum, C.; Myers, R.M.; Brown, M.; Li, W.; et al. Model-based analysis of ChIP-Seq (MACS). Genome Biol. 2008, 9, R137.
macs3	GSE59703	3.0.0b3	
Unibind	promoters of DSG	2.0	Puig, R.R., Boddie, P., Khan, A. *et al.* UniBind: maps of high-confidence direct TF-DNA interactions across nine species. *BMC Genomics* **22**, 482 (2021). https://doi.org/10.1186/s12864-021-07760-6

Supplementary Table 2. Bioinformatic tools and version numbers.

Target	Forward primer	Reverse primer
PEMT-C	GTTCCCAGAGGGCAGACTTC	GGGTCTTGTGTTCCCATCGT
PEMT-L	GGAGCCGAGGTAACGAACAG	CCGGGTCATAACGCAGAAGT
PEMT	TCCGCTCTACTGGAATGTGG	TTGAAGGGGAACACGGTCAC
PPARG1	TGACCAGAAGCCTGCATTTCT	AAGGCTGACTCTCGTTTGAG
PPARG2	AAGCGATTCCTTCACTGATACAC	CTTCCATTACGGAGAGATCCAC
SF3A1	AGGGTCCAGTGTCCATCAAA	AGAGACCTGGTCCGTGAGTG

Supplementary Table 3. qPCR primer sequences
